# Practical Points

**Published:** 1894-12-15

**Authors:** 


### PRACTICAL POINTS.

*Hospital Ships.—M. D. writes: Can you kindly inform me where I can obtain the latest information on the subject, not only as to hospital ships in this country but also in foreign countries?*

Information as to hospital ships is not extensive, as they are very little used, and are going out of fashion, except for isolation purposes. The Metropolitan Asylums Board, Norfolk House, Norfolk Street, Strand, have several ships moored on the Thames at Long Reach, and a regular system of river carriage for patients. There is also a floating cholera hospital on the Tees. The hospital ship at Cardiff is, we believe, to be removed on shore. Any further information concerning foreign countries would be procurable, if at all, from the ministers of Marine and War at their respective capitals. It is, however, doubtful if the result would be likely to compensate for the trouble of making inquiries, except perhaps in the case of the United States of America, where there is a fairly organised system of transport by hospital ships.

